# Effect of IL-17A on the Migration and Invasion of NPC Cells and Related Mechanisms

**DOI:** 10.1371/journal.pone.0108060

**Published:** 2014-09-22

**Authors:** Lixin Wang, Ruixia Ma, Zhaopeng Kang, Yupeng Zhang, Hongcheng Ding, Weina Guo, Qing Gao, Min Xu

**Affiliations:** 1 Department of Otolaryngology head and neck surgery, Second Hospital of Xi'an Jiaotong University, Xi'an, China; 2 Department of Otolaryngology head and neck surgery, Affiliated People's Hospital of Hubei Medical University, Shiyan, China; 3 Department of Otolaryngology head and neck surgery, Hospital Affiliated to Ningxia Medical University, Yin chuan, China; 4 Department of Obstetrics and Gynecology, the Second Affiliated Hospital, Medical School of Xi'an Jiaotong University, Xi'an, P. R. China; National Cancer Center, Japan

## Abstract

In carcinogenesis, inflammasomes may play contradictory roles through facilitating anti-tumor immunity or inducing oncogenic factors. Their function in cancer remains poorly characterized. In this study, we explored the effect of interleukin-17A (IL-17A) on the migration and invasion activity of nasopharyngeal carcinoma (NPC) cell lines and account for related mechanisms. Our results revealed that exogenous IL-17A promoted cell migration and invasion significantly in both NPC-039 and CNE-2Z cell lines. In addition, the expression of matrix metalloproteinase-2 (MMP-2)/-9 and Vimentin could be elevated by IL-17A stimulation; meanwhile the expression of E-cadherin was decreased. The results also show that IL-17A could activate the p38 signaling pathway in IL-17A-stimulated NPC-039 and CNE-2Z cell lines. Combining treatment with a p38 inhibitor (SB203580) resulted in decreased invasion capabilities of NPC-039 and CNE-2Z cell lines. SB203580 also inhibited the expression of MMP-2/-9 and increased the expression of E-cadherin in IL-17A-stimulated NPC-039 and CNE-2Z cell lines. IL-17A also could activate NF-κB in NPC-039 and CNE-2Z cell lines. In summary, our data show that IL-17A promote the cell migration and invasion of NPC cells. The effect of IL-17A on cell migration and invasion may be mediated via regulation of the expression of MMP-2/-9 and epithelial-mesenchymal transition (EMT) via p38-NF-κB signaling pathway. Thus, IL-17A or its related signaling pathways may be a promising target for preventing and inhibiting NPC metastasis.

## Introduction

Nasopharyngeal carcinoma (NPC) shows the highest metastasis features among head and neck cancers. Distant metastasis remains the main barrier to the treatment in NPC patients. Up to 75% of NPC patients occur metastasis to the neck lymph nodes, which represents an adverse prognostic factor of the NPC [1].

Metastasis is a complex process, including reduction of tumor cell adhesion, degradation of extracellular matrix (ECM), enhancement of cell motility, and promotion of neo-vascularization [2]. During the process of metastasis, MMPs play important roles via degrading ECM [2]. Epithelial-mesenchymal transition (EMT) also plays an important role in tumor metastasis [3]. During the process of EMT, cancer cells will lose epithelial cell markers and meanwhile acquire mesenchymal markers [4]. Through EMT, the motility ability of cells was enhanced and subsequently made metastasis possible.

Recently, interleukin-17A (IL-17A) has been also frequently observed in many cancers such as ovarian cancer [5], breast cancer [6], gastric cancer [7], and hepatocellular carcinoma [8]. IL-17A was also found to be correlated with the invasion of cancer cells [9], [10]. But up to now, the role of IL-17A in NPC progression is not fully illuminated.

In the study, we attend to analyse the effect of IL-17A on the migration and invasion of NPC cells. We found that IL-17A could increase cell motility by regulating MMPs and EMT via activating p38- NF-κB signaling pathway.

## Materials and Methods

### Reagents

Anti E-cadherin and Anti Vimentin were purchased from Abcam. Fetal bovine serum (FBS), penicillin, streptomycin and Dulbecco's modified Eagle's medium (DMEM) were ordered from Hyclone. Anti-MMP-2, MMP-9, NF-κB p50, NF-κB p65, NF-κB p52, NF-κB RelB, NF-κB c-Rel anti-p38 and anti-p-p38 antibodies were purchased from Cell Signaling. Anti-Histone H1antibody was purchased from Santa Crus.

### Cell culture

Nasopharyngeal carcinoma-derived cell line NPC-039 was cultured in Dulbecco's modified Eagle's medium (DMEM) supplemented with 10% fetal bovine serum (FBS) [11]. CNE-2Z is one of nasopharyngeal carcinoma cell lines with low differentiation and high transfer which could be conductive to the study in migration and invasion in nasopharyngeal carcinomca cells. Poorly differentiated human CNE-2Z cell lines were obtained from Zhongshan University and cultured in the current laboratory [12].

### Assessment of cell apoptosis

Apoptotic and/or necrotic cells were evaluated by Annexin V binding and propidium iodide (PI) uptake using an Annexin V-FITC/PI kit as previously described [13]. Briefly, NPC-039 cells were plated at a density of 5×10^4^ cells per well into 6-well plates for 12 h. The cells were treated with various concentrations of IL-17A (0 and 50 ng/ml) and incubated at 37°C for 24 h. The cells were washed with cold PBS and resuspended in Annexin V binding buffer. The cells were stained with Annexin V-FITC for 15 min, washed, and then stained with PI. The samples were analyzed by flow cytometer with CellQuest software.

### Wound healing assay

Cell migration was assessed by a scratch wound-healing assay. Cells were cultured in 6-well plate until confluent rate reached 70–80% and then treated with or without IL-17A (R&D System, Minneapolis, MN) (1, 10 and 50 ng/ml). The cell layer was wounded using a sterile tip and the spread of wound closure was observed and photographed.

### Cell invasion assay

Invasion assay was performed with 24-well BioCoat Matrigel Invasion Chambers (Becton Dicknson, Bedford, MA) according to the manufacturer's instructions. After cultured in medium with different concentrations of IL-17A (0, 1, 10 and 50 ng/ml), cells were seeded into inner well and cultured for 24 h, and then cells that invaded through the Matrigel was fixed, dyed and measured.

### NF-κB activity ELISA

DNA binding activity of NF-κB p50, p65, p52, RelB, and c-Rel in IL-17A treated cells was detected using TransAM NF-κB ELISA (Active Motif). NF-κB activity ELISA was carried out as described earlier [14].

### Western blot analysis

After treated with various concentrations of IL-17A or SB203580, 2×10^5^ cells were suspended in 100 µl of lysis buffer (40 mmol/l Tris-HCl, 1 mmol/l EDTA, 150 mmol/l KCl, 100 mmol/l NaVO3, 1% Triton X-100, 1 mmol/l PMSF, pH 7.5). Nuclear lysates from cultured NPC cells were harvested with NucBusterTM Protein Extraction Kit (Novagen, Germany) according to manufacturer's instructions. The proteins (70 µg) were separated on 8% or 12% SDS-polyacrylamide gel electrophoresis and transferred onto PVDF membranes. The membranes were subsequently blocked in defatted milk (5% in Tris-buffered saline with TWEEN-20 (TBST) buffer) at 37°C for 1 h to block non-specific binding and then incubated overnight with antibodies against E-cadherin, Vimentin, p38, p-p38, MMP-2, MMP-9, Histone H1 and β-actin in TBST containing 5% defatted milk at 4°C. Then membranes were incubated with a horseradish peroxidase goat anti-mouse or anti-rabbit IgG for 1 h at room temperature. The bands were detected by an enhanced chemiluminescence kit (Amersham, ECL Plus, Freiburg, Germany) and exposed by autoradiography. The densitometric analysis was done using Image J software (GE Healthcare, Buckinghamshire, UK) and expressed as arbitrary units (a. u.).

### Statistical analysis

All data were expressed as the means ± SD. The statistical analysis was carried out using the SPSS 16.0 software (SPSS Inc., Chicago, Illinois, USA) to evaluate statistical differences. Student's t-test was used for comparisons between two groups and one-way or two-way analysis of variance was used to analyze statistical differences between groups under different conditions. *P*<0.05 was considered to be statistically significant. All statistical tests were two sided. We performed correlation analysis by Z test.

## Results

### IL-17A increased cell motility in NPC cells

**Figure 1:**
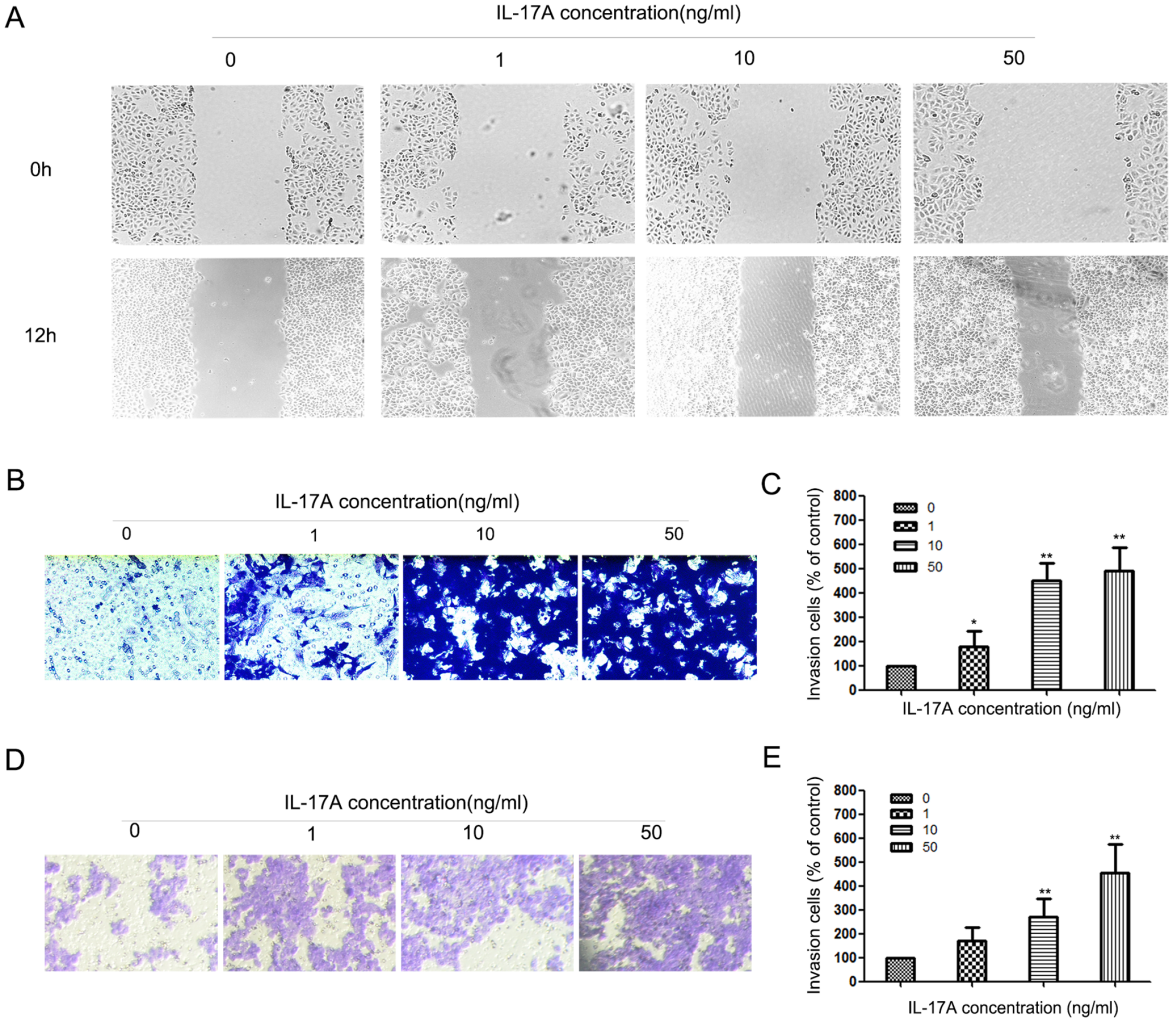
IL-17A promotes NPC cell migration and invasion. (A) IL-17A treated NPC-039 cells showed higher motility in a wound-healing assay, compared with cells without IL-17A treatment. (B) Effect of IL-17A on NPC-039 cells invasion was detected by transwell assay. Representatives of cells migrated through Matrigel-coated transwell were shown. (C) Total invasive cell number in each chamber was summarized as a percentage of control. (D) Effect of IL-17A on CNE-2Z cells invasion was detected by transwell assay. Representatives of cells migrated through Matrigel-coated transwell were shown. (E) Total invasive cell number in each chamber was summarized as a percentage of control. Values represent the means ± SD of three independent experiments performed in triplicate. *p<0.05 and **p<0.01 compared with the control group.

Wound healing and matrigel invasion assays were employed to test the effect of IL-17A on cell motility. The results of wound healing show that migrations of NPC-039 cells were enhanced by IL-17A (Figure 1A). The results of transwell assay showed that the invasions of NPC-039 cells were enhanced by IL-17A too (Figure 1B and C). We also found that IL-17A could increase the invasion of CNE-2Z cells (Figure 1D and E).

### IL-17A up-regulated MMP-2, MMP-9 and Vimentin expression and down-regulated E-cadherin expression in NPC-039 and CNE-2Z cell lines

**Figure 2:**
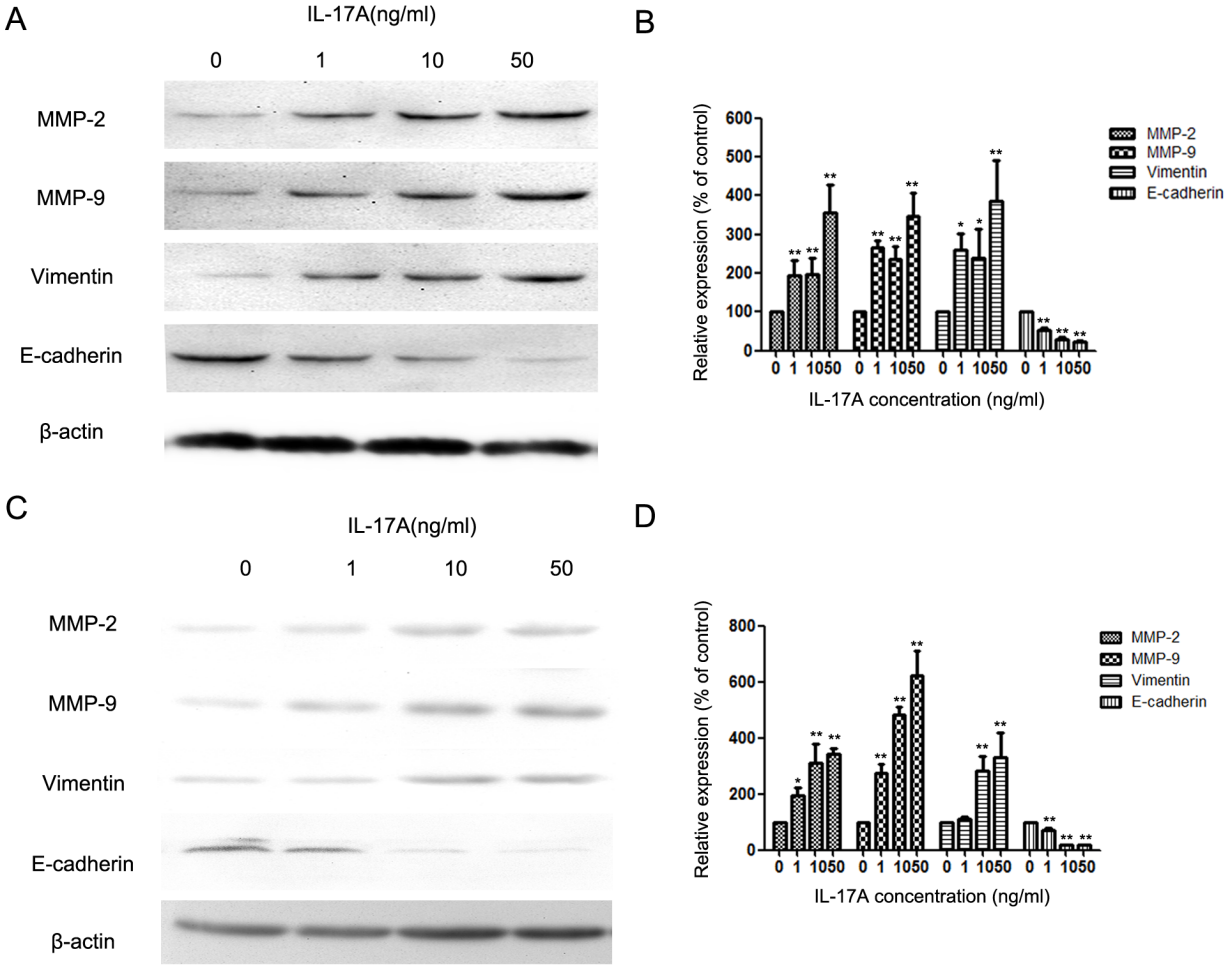
IL-17A promotes the expressions of MMP-/-9 and Vimentin suppresses the expressions of E-cadherin in NPC-039 and CNE-2Z cells. (A) Expressions of MMP-2/-9, Vimentin and E-cadherin in NPC-039 cells were compared by western blotting between cells treated with different concentrations of IL-17A (0, 1, 10 and 50 ng/ml) for 24 hours. (B) Quantification of the protein levels of MMP-2/-9, Vimentin and E-cadherin in NPC-039 cells. (C) Expressions of MMP-2/-9, Vimentin and E-cadherin in CNE-2Z cells were compared by western blotting between cells treated with different concentrations of IL-17A (0, 1, 10 and 50 ng/ml) for 24 hours. (D) Quantification of the protein levels of MMP-2/-9, Vimentin and E-cadherin in CNE-2Z cells. Values represent the means ± SD of three independent experiments performed in triplicate. *p<0.05 and **p<0.01 compared with the control group.

MMP-2 and MMP-9 play important roles in cancer metastasis [2], and IL-17A can affect the expression of MMP-2 and MMP-9 [15]. After treating with IL-17A the expression of MMP-2 and MMP-9 in NPC-039 cells was increased (Figure 2A and B). EMT is closely correlated with the invasion of cancer cells, through which the expression of E-cadherin and Vimentin will change. IL-17A could promote the progression of EMT [16]. In the study, we found that IL-17A could down-regulate the expression of E-cadherin and up-regulate the expression of Vimentin (Figure 2A and B). Similar effect of IL-17A on CNE-2Z cell lines was also found (Figure 2C and D).

### IL-17A up-regulated MMP-2, MMP-9 and Vimentin expression and down-regulate E-cadherin expression via activating p38 signaling pathway

**Figure 3:**
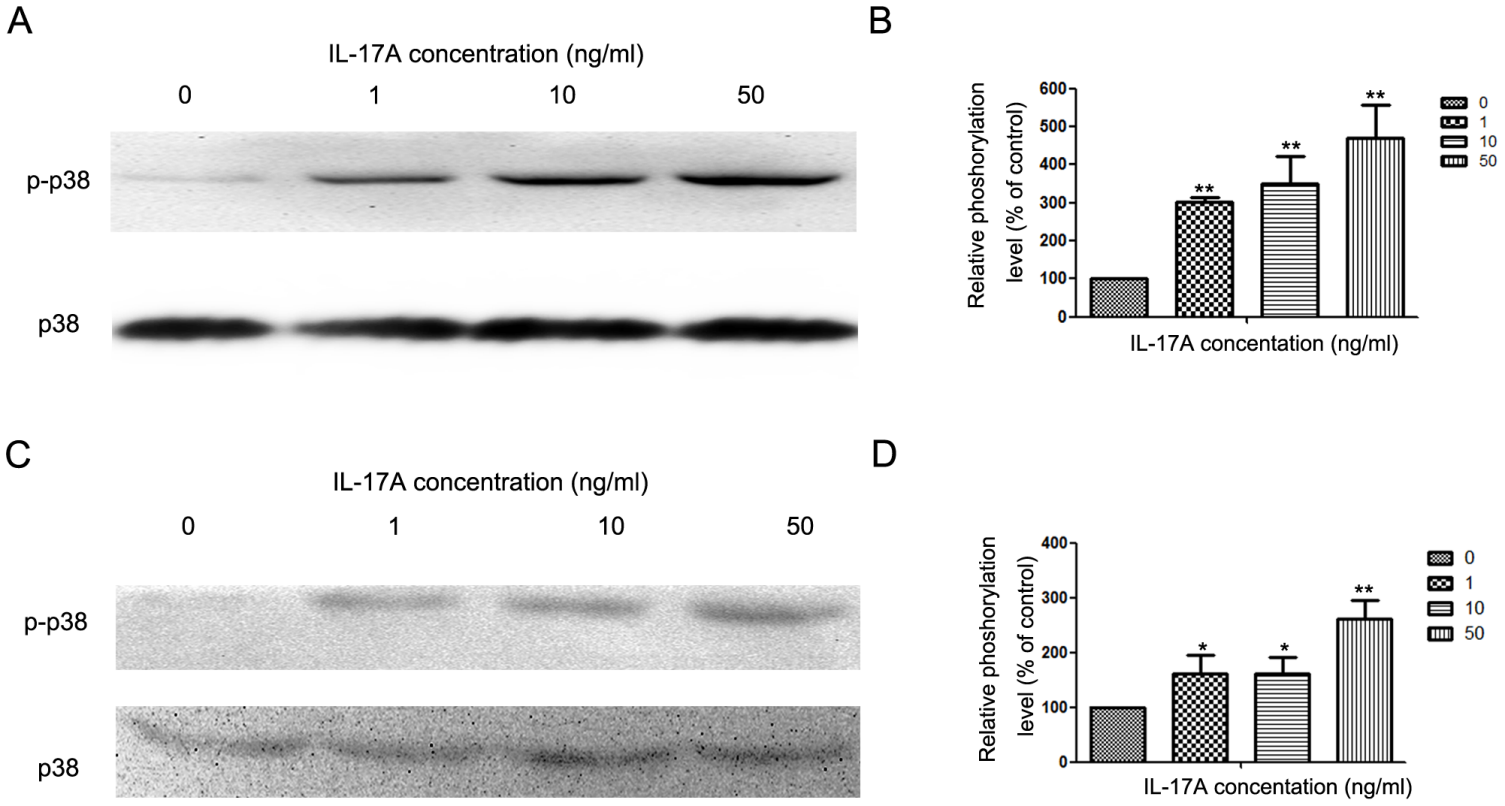
IL-17A activated p38 signaling pathway in NPC cells. (A) Western blotting analysis was used to detect p38 and p-p38 expression in NPC-039 cells treated with IL-17A (0, 1, 10 and 50 ng/ml) at indicated time points. (B) Quantification of the protein levels of p38 and p-p38 in NPC-039 cells. (C) Western blotting analysis was used to detect p38 and p-p38 expression in NPC-039 cells treated with IL-17A (0, 1, 10 and 50 ng/ml) at indicated time points in CNE-2Z cells. (D) Quantification of the protein levels of p38 and p-p38 in CNE-2Z cells. Values represent the means ± SD of three independent experiments performed in triplicate. *p<0.05 and **p<0.01 compared with the control group.

In the study, we found that IL-17A induces phosphorylation of p38 in NPC cells, which is in accordance with earlier findings [17], [18]. After treating with IL-17A, the phosphorylation level of p38 signaling pathway was increased (Figure 3A and B). In CNE-2Z cell lines, IL-17A could also increase the phosphorylation level of p38 (Figure 2C and D).

**Figure 4:**
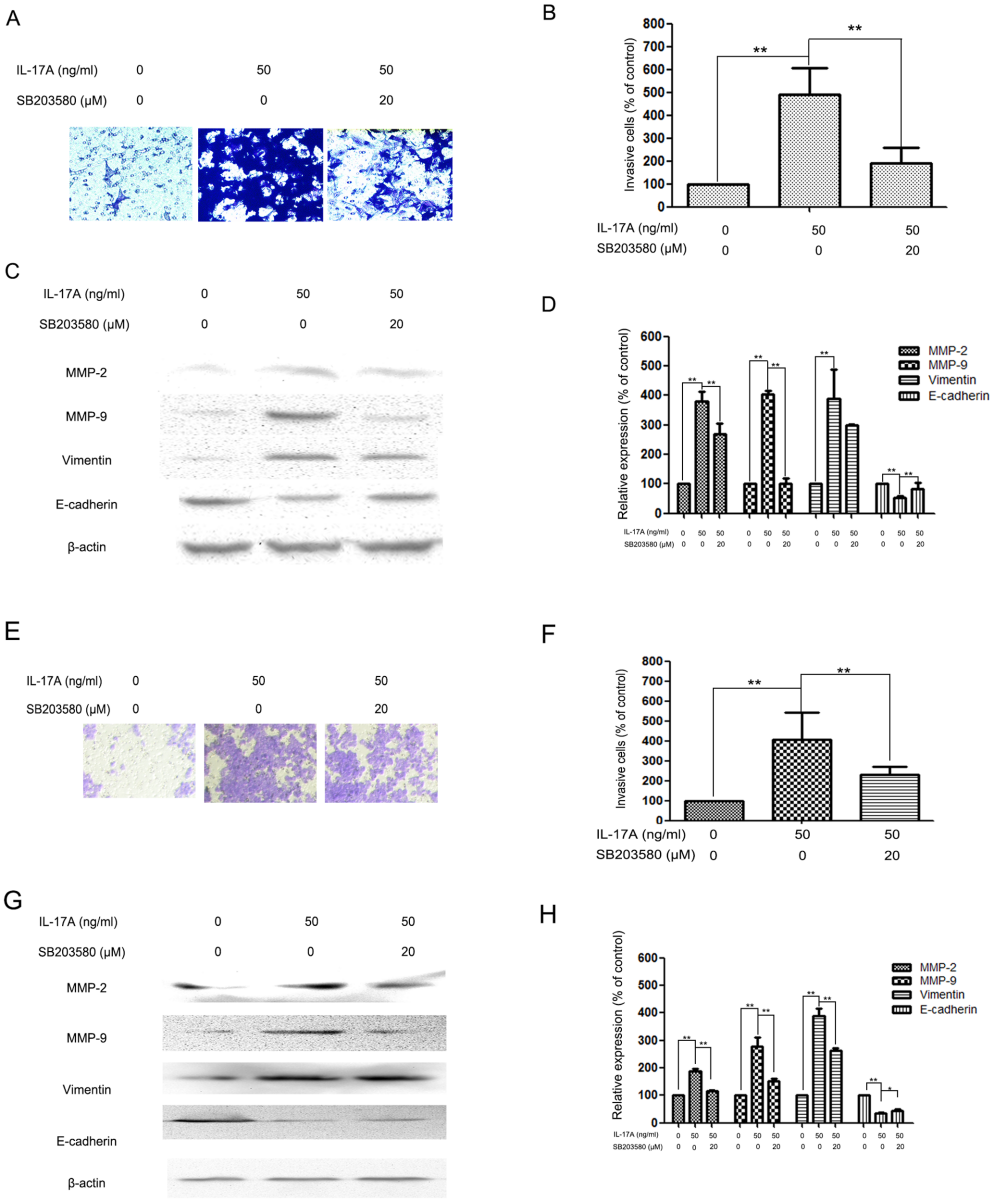
Effects of the p38 inhibitor and IL-17A on cell invasion and MMP-2, MMP-9, Vimentin and E-cadherin expressions in NPC-039 and CNE-2Z cells. (A) NPC-039 cells were pretreated with SB203580 (20 µM) for 30 min and then incubated in the presence or absence of IL-17A (50 ng/ml) for 24 h. Cellular invasiveness was measured using the transwell invasion assay. (B) The percent invasion rate in NPC-039 cells was expressed as a percentage of control. (C, D) NPC-039 cells were treated and then subjected to western blotting to analyze the protein levels of MMP-2/-9, Vimentin and E-cadherin. (E) CNE-2Z cells were pretreated with SB203580 (20 µM) for 30 min and then incubated in the presence or absence of IL-17A (50 ng/ml) for 24 h. Cellular invasiveness was measured using the transwell invasion assay. (F) The percent invasion rate in CNE-2Z cells was expressed as a percentage of control. (G, H) CNE-2Z cells were treated and then subjected to western blotting to analyze the protein levels of MMP-2/-9, Vimentin and E-cadherin. Values represent the means ± SD of three independent experiments performed in triplicate. *p<0.05 and **p<0.01 compared with the control group.

In order to further research whether the inhibitory effect of IL-17A on cell invasion and MMP-2/9 expression and EMT was correlated with activation of the p38 signaling pathway, NPC cells were pretreated with a p38 inhibitor (SB203580, 20 µM) for 30 min and then incubated in the presence or absence of IL-17A (50 ng/ml) for 24 h. The results show that treatment with SB203580 significantly inhibited cell invasion (Figure 4A,4B, 4E and 4F) and reduced MMP-2/-9 protein expression (Figure 4C and 4D). Meanwhile, the expression of E-cadherin was increased (Figure 4C, 4D, 4G and 4H).

### NF-κB contributes to IL-17-mediated NPC cells invasion

**Figure 5:**
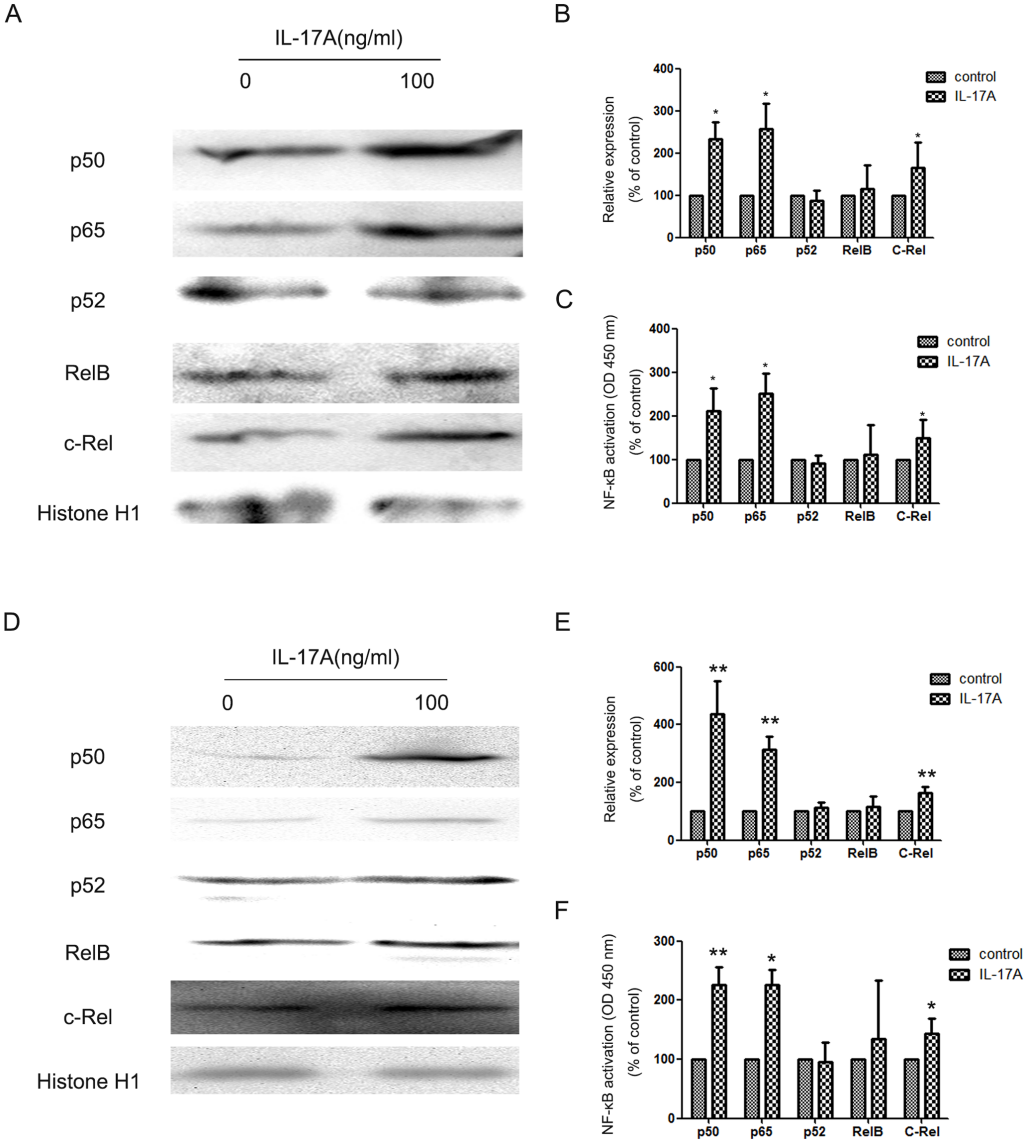
IL-17A activated NF-κB NPC in NPC cells. (A) Western blotting analysis was used to detect nuclear p52, p50, p65, c-Rel and RelB expression in NPC-039 cells treated with IL-17A. (B) Quantification of the protein levels of nuclear p52, p50, p65, c-Rel and RelB in NPC-039 cells. (C) The DNA-binding capacity of NF-κB in NPC-039 cells was measured using TransAM NF-κB ELISA. (D) Western blotting analysis was used to detect nuclear p52, p50, p65, c-Rel and RelB expression in CNE-2Z cells treated with IL-17A. (E) Quantification of the protein levels of nuclear p52, p50, p65, c-Rel and RelB in CNE-2Z cells. (F) The DNA-binding capacity of NF-κB in CNE-2Z cells was measured using TransAM NF-κB ELISA. Values represent the means ± SD of three independent experiments performed in triplicate. *p<0.05 compared with the control group.

NF-κB has been reported as a downstream target of IL-17A in many cells [15], [19], [20], which is able to promote MMP-2/-9 expressions [15], [21]. And IL-17A was also reported to increase the expression of MMPs via activating NF-κB pathway in many cells [15], [21]. NF-κB also palyes an important role in the induction and maintenance of EMT [22], [23]. So we next detected whether the promoting effect of IL-17A on MMP-2/-9 expressions in NPC cells was also by activating NF-κB or not. The result showed that the level of p50, p65, and c-Rel in nuclei was dramatically elevated in NPC cells after IL-17A treatment (Figure 5A, 5B, 5D and 5E). The DNA-binding capacity of NF-κB in NPC cells was measured in NPC cells (Figure 5C and 5F). The result demonstrated that IL-17A induced MMP-2/-9 expression in NPC via NF-κB activation.

## Discussion

Metastasis is the main obstacle in the current clinical management of NPC. Preventing, predicting, and inhibiting NPC metastasis is therefore critical for further improving the survival rate of patients with NPC. IL-17A has been found to be closely correlated with the metastasis of cancer [9], [24], [25]. Up to now, the effect of IL-17A on NPC metastasis and related mechanisms are still unclear.

In the study, we found that IL-17A could promote the migration and invasion of NPC cells. Metastasis is one of the leading causes of cancer-related death among NPC patients. Degradation of the ECM of blood or lymph vessels is critical to metastasis, loss of the ECM allows cancer cells to invade the lymphatic or blood system and spread to other tissues and organs [2]. MMPs, especially MMP-2 and MMP-9, are responsible for breaking down the ECM [2], [26]. IL-17A was reported to promote the invasion of cancer cells via up-regulating the expression of MMP-2/-9 [15], [27], [28]. Our results showed that IL-17A elevated the expression of MMP-2/-9. The results suggest the pro-metastasis effect of IL-17A on NPC is correlated with the regulation of expression of MMP-2/-9.

Carcinoma invasion requires tumor cells to gain the ability to degrade the underlying basement membrane and ECM. The EMT program is involved in this process through up-regulation of various matrix degradation enzymes by the EMT core regulators [29]. Up to now, EMT is a process characterized by loss of cell adhesion, reduction of E-cadherin expression but increase in cell motility. During EMT, E-cadherin gene transcriptional repression, promoter methylation, and protein phosphorylation and degradation have all been observed in response to various inducing signals [29]. Meanwhile, mesenchymal markers' expression increase, such as Vimentin. Vimentin has been found to be overexpressed in various epithelial cancers, including prostate cancer, gastrointestinal tumors, breast cancer, malignant melanoma and lung cancer [30]. The high expression of Vimentin in cancer is associated with accelerated tumor growth invasion and poor prognosis [31]. In the study, we found that IL-17A could down-regulate the expression of E-cadherin and up-regulate the expression of Vimentin.

p38 singaling pathway is reported to play important roles in the metastasis of NPC via regulating MMPs [32]. During the process of EMT, p38 signaling pathway also plays an important role [33]. p38 singaling pathway has also been reported as a downstream target of IL-17A in many cells [34], [35]. To further explore the possible mechanism(s) of IL-17A in the inhibition of NPC invasion, we have detected the levels of phosphorylation of p38 in NPC-039 cells. The results demonstrated that the phosphorylation of p38 in cells treated with IL-17A was significantly increased relative to that in control cells. SB203580 abolished the promoting effect of IL-17A on the invasion of NPC-039 cells, meanwhile down-regulated the expression MMP-2/-9 and Vimentin, and up-regulated the expression of E-cadherin.

NF-κB has been reported as a downstream target of IL-17A in many cells [15], [19], [20], which is able to promote MMP-2/-9 expressions [15], [21]. And IL-17A was also reported to increase the expression of MMPs via activating NF-κB pathway in many cells [15], [21]. NF-κB also plays an important role in the induction and maintenance of EMT [22], [23]. In the study, we found that IL-17A could elevate the level of p50, p65, and c-Rel in nuclei was dramatically in NPC cells after IL-17A treatment.

In conclusion, our findings illustrated that IL-17A was able to promote the migration and invasion of NPC cells by activating p38- NF-κB signaling pathway, which subsequently regulated the expression of MMP-2/-9 and EMT. Further characterization of the effect of IL-17A on NPC invasion and metastasis may lead to the identification of new diagnostic markers and therapeutic targets.

## Supporting Information

Figure S1 **The effect of IL-17A on the apoptosis of NPC-039 cells.** The results showed that IL-17A did not affect the apoptosis of NPC-039 cells.
(TIF)
