# Supplementary figures and images for: Effect of IL-17A on the Migration and Invasion of NPC Cells and Related Mechanisms
Source: PLoS One. 2014 Sep 22;9(9):e108060. doi: 10.1371/journal.pone.0108060 (PMC4171532; doi:10.1371/journal.pone.0108060)

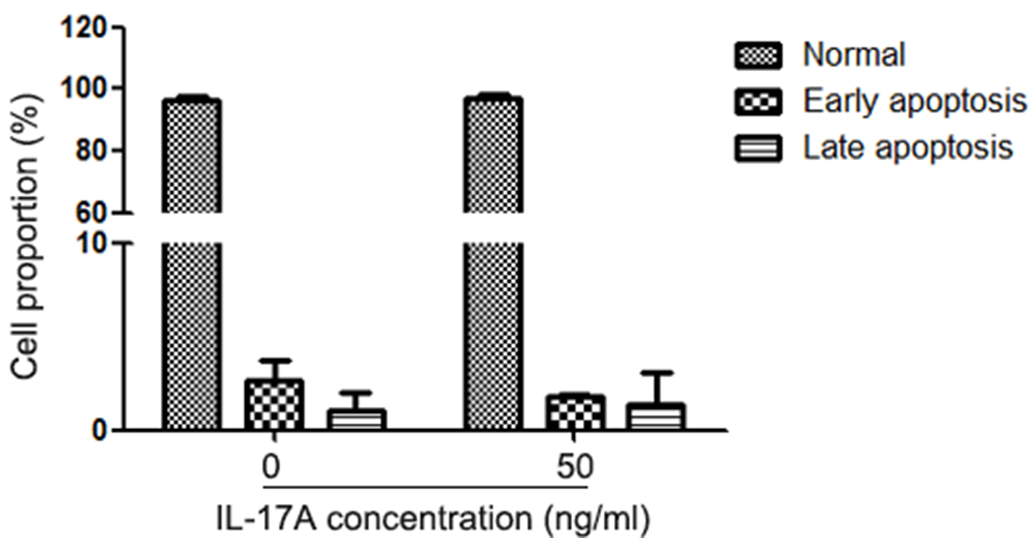

Supplement: Figure S1 — The effect of IL-17A on the apoptosis of NPC-039 cells. The results showed that IL-17A did not affect the apoptosis of NPC-039 cells. (TIF) [file pone.0108060.s001.tif]
